# Upconversion-based chiral nanoprobe for highly selective dual-mode sensing and bioimaging of hydrogen sulfide in vitro and in vivo

**DOI:** 10.1038/s41377-024-01539-6

**Published:** 2024-08-01

**Authors:** Yang Lu, Xu Zhao, Dongmei Yan, Yingqian Mi, Peng Sun, Xu Yan, Xiaomin Liu, Geyu Lu

**Affiliations:** 1 State Key Laboratory of Integrated Optoelectronics, College of Electronic Science and Engineering, Jilin University, 130012 Changchun, China; 2 Department of Immunology, College of Basic Medical Sciences, Jilin University, 130021 Changchun, China

**Keywords:** Nanoparticles, Biophotonics

## Abstract

Chiral assemblies have become one of the most active research areas due to their versatility, playing an increasingly important role in bio-detection, imaging and therapy. In this work, chiral UCNPs/Cu_x_OS@ZIF nanoprobes are prepared by encapsulating upconversion nanoparticles (UCNPs) and Cu_x_OS nanoparticles (NPs) into zeolitic imidazolate framework-8 (ZIF-8). The novel excited-state energy distribution-modulated upconversion nanostructure (NaYbF_4_@NaYF_4_: Yb, Er) is selected as the fluorescence source and energy donor for highly efficient fluorescence resonance energy transfer (FRET). Cu_x_OS NP is employed as chiral source and energy acceptor to quench upconversion luminescence (UCL) and provide circular dichroism (CD) signal. Utilizing the natural adsorption and sorting advantages of ZIF-8, the designed nanoprobe can isolate the influence of other common disruptors, thus achieve ultra-sensitive and highly selective UCL/CD dual-mode quantification of H_2_S in aqueous solution and in living cells. Notably, the nanoprobe is also capable of in vivo intra-tumoral H_2_S tracking. Our work highlights the multifunctional properties of chiral nanocomposites in sensing and opens a new vision and idea for the preparation and application of chiral nanomaterials in biomedical and biological analysis.

## Abstract (web summary)

The schematic of the preparation process of chiral UCNPs/Cu_x_OS@ZIF nanoprobe, in vitro highly selective UCL/CD dual-mode sensing for hydrogen sulfide, and in vivo bioimaging.

## Graphical abstract

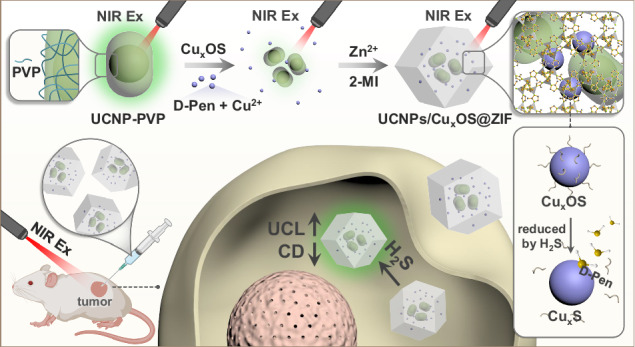

## Introduction

Chirality is ubiquitous in nature. A chiral object cannot be overlapped with its mirror image^1–3^. Most biological macromolecules are chiral, such as DNA^4,5^, amino acids^6,7^, peptides^8^, and proteins^9,10^. Owing to the excellent safety and biocompatibility of these natural chiral compounds, numerous artificial chiral nanomaterials have been increasingly constructed, including single chiral nanoparticles^11,12^, chiral nanocomponents^13–15^ and two-dimensional (2D) chiral nanofilms^16,17^. As one of the most representative optical properties of these chiral nanomaterials, circular dichroism (CD) is an ideal and powerful sensing technology with the potential for non-destructive cell analysis. In recent years, many excellent research results have been reported on biosensing using CD spectral signals, which exhibit higher sensitivity compared to other analytical methods. Chiral Cys-capped MoO_2_ NPs and chiral DNA-modified Ag NPs were designed for the detection of metal ions, which possessed comparable to or even better sensing performance than previously reported surface-enhanced Raman scattering (SERS), colorimetric strategy, fluorescent methods, and electrochemical-based sensors^18,19^. Despite the excellent sensitivity of CD-based chiral sensors, the time-consuming process of sample preprocessing, signal acquisition, and analysis makes it difficult to perform real-time on-site sensing and analysis of cells and living tissues, which is critical for biomonitoring in rapidly changing physiological environments.

In order to have a broader application prospect in the biological field, scientists have combined some other materials with the chiral material to give such chiral nanocomposites more diverse bio-functional characteristics. Combining chiral signals with other signals such as fluorescence or Raman, these chiral nanocomposites can be used for real-time multimodal detection of small biological changes and disease markers^20^. Chiral composites with imaging properties such as magnetic resonance (MR) are used to visualize and monitor biological processes for safe, non-invasive disease diagnosis^21^. Besides, there is also a class of chiral inorganic nanomaterials decorated with molecules compatible with biomolecules or ligands, which can specifically target disease-related receptors for photodynamic and photothermal therapy^22,23^. However, some chiral nanocomposites assembled by electrostatic adsorption or other methods have poor structural stability and are prone to dissociation and destruction in complex physiological environment, leading to deviations in performance. In addition, some chair composite sensing materials are difficult to distinguish interferents with similar properties to the analyte, resulting in poor detection selectivity^21,24^. Therefore, the development of chiral composite nanomaterials with stable structure and excellent performance to meet the needs of biomedical diagnosis and detection remains a challenging.

**Fig. 1:**
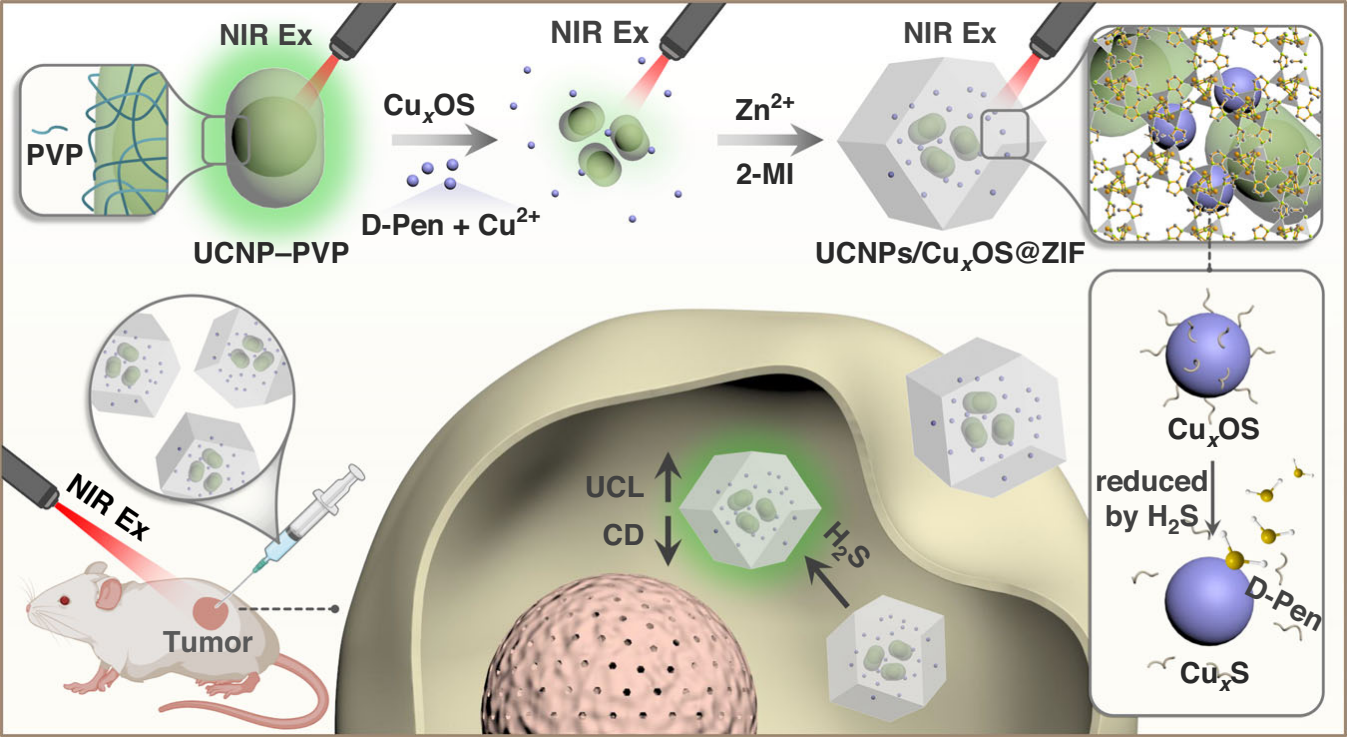
The preparation process and H_2_S biosensing application of UCNPs/Cu_*x*_OS@ZIF nanoprobe. The UCNPs/Cu_x_OS@ZIF nanoprobes are prepared by coating UCNPs and Cu_x_OS with ZIF-8. The UCL signal and CD signal of the nanoprobe will change respectively in the presence of H_2_S, so as to achieve dual-mode biosensing in cells and in vivo

In this work, we constructed UCNPs/Cu_x_OS@ZIF nanoprobes for UCL/CD dual-mode real-time detection of H_2_S in vitro and in vivo. The preparation process and work principle of the UCNPs/Cu_x_OS@ZIF nanoprobes are illustrated in Fig. 1. The designed NaYbF_4_@NaYF_4_: 20% Yb^3+^, 2% Er^3+^ UCNPs fluorescent donors maximized the energy donation to the acceptors by employing 100% activator Yb^3+^ ions in the nucleus to harvest NIR excitation energy and by confining the emitter Er^3+^ ions to the surface layer to shorten the energy transfer distance. Chiral Cu_x_OS NPs were used as the energy acceptors and CD sources. Then zeolitic imidazolate framework-8 (ZIF-8) was used as a shell to encapsulate UCNPs and chiral Cu_x_OS to construct the nanocomposite. The small aperture of ZIF-8 can effectively exclude some disruptors chemically similar to H_2_S (such as GSH, L/D-Cys and L-Lys), thereby directly improving the specificity of detection. The reduction of Cu_x_OS by H_2_S led to changes in Cu_x_OS absorption and CD signals, allowing the designed nanoprobes to produce UCL and CD signals specifically in response to H_2_S. Taking advantages of UCNPs in bio-imaging^25^, we achieved ultra-sensitive and highly selective quantification of H_2_S in aqueous solution and in living cells, as well as intratumoral imaging of H_2_S in vivo. The construction of the UCNPs/Cu_x_OS@ZIF dual-mode nanoprobe makes chiral sensing a more favorable tool in bioassays and provides a new idea for the application of multi-functional chiral nanomaterials in biomedicine.

## Results

### Construction and characterization of nanocomposite probes

**Fig. 2:**
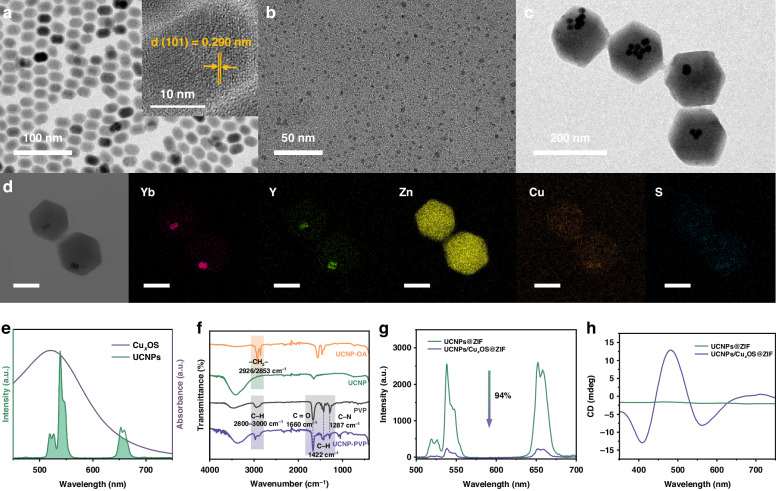
Morphological characterization and properties. Transmission electron microscopy (TEM) images of (**a**) NaYbF_4_@NaYF_4_:Yb,Er core/shell nanoparticles. Inset shows the high-resolution TEM image of the core/shell nanoparticles. **b** Cu_x_OS nanoparticles and **c** UCNPs/Cu_x_OS@ZIF nanoprboes. **d** The corresponding elemental mapping images of UCNPs/Cu_x_OS@ZIF. Scale bar = 100 nm. **e** Absorption spectrum of Cu_x_OS and the UCL spectrum of UCNPs. **f** Fourier transform infrared spectroscopy (FT-IR) of UCNP-OA, UCNP, PVP and UCNP-PVP. Changes of (**g**) UCL spectra and (**h**) CD spectra after Cu_x_OS NPs were added into nanoprboes

The designed NaYbF_4_@NaYF_4_:Yb, Er UCNPs were obtained through a previous solvothermal method^26^. Figures 2a and S1 were the transmission electron microscopy (TEM) images of synthesized UCNPs. The core (NaYbF_4_) nanoparticles were spherical particles with good uniform size and dispersion, with an average size of 16.38 ± 0.78 nm. After coating the NaYF_4_: Yb, Er luminescent shell, the size increased to (18.08 ± 0.19) * (24.91 ± 1.57) nm, showing an ellipsoid shape (Fig. S2). The illustration in Fig. 2a showed the lattice spacing of (101) crystal planes was 0.290 nm, which was consistent with the standard hexagonal NaYF_4_:Yb, Er. The X-ray diffraction (XRD) results also confirmed that both core and core/shell nanoparticles had a pure hexagonal phase (Fig. S3). The conventional NaYF_4_: Yb, Er UCNP donor showed low efficiency and poor sensitivity because only partial activators in UCNPs with a suitable distance from the energy acceptor can activate the fluorescence resonance energy transfer (FRET) process (Fig. S6a, c). In contrast, the excited-state energy distribution-modulated upconversion donor NaYbF_4_@NaYF_4_:Yb, Er can obtain bright upconversion green luminescence (Figs. S4, S5) and higher energy transfer efficiently by concentrating the 100% sensitizer Yb^3+^ ions in the core to maximize the absorption of NIR excitation energy and confining the emitter Er^3+^ ions in the outer layer to shorten the energy transfer distance (Fig. S6b, d)^27^.

The acceptors Cu_x_OS NPs were synthesized under alkaline conditions. A brown Pen-Cu-Pen complex was first generated via the coordination of Cu^2+^ ions and sulfhydryl groups (-SH) in D-penicillamine (D-Pen), then the chiral Cu_x_OS NPs were crystallized via the reduction of hydroxylamine hydrochloride. We first optimized the amount of D-Pen and Cu^2+^ ions during the synthesis of Cu_x_OS. By testing the absorption spectra and CD spectra of Cu_x_OS synthesized at different D-Pen: Cu^2+^ ratios, we found that the best absorption and CD spectral performance of Cu_x_OS was achieved with a D-Pen: Cu^2+^ ratio of 3:1 (Figs. S7, S8). The Cu_x_OS NPs synthesized under this ratio (D-Pen: Cu^2+^ = 3:1) had good dispersion and an average size below 5 nm (Figs. 2b and S9). Because of the coordination of Cu^2+^ ions and -SH during the formation of Cu_x_OS, the peak of -SH in D-Pen at 2550 cm^−1^ in the Fourier transform infrared spectrum (FT-IR) disappeared after the formation of Cu_x_OS (Fig. S10)^28^. The chiroptical activity of the D-Pen and Cu_x_OS was then investigated. The original CD signal of D-Pen at 240 nm was transformed into three new signals at 408 nm, 481 nm, and 569 nm, respectively, which were Cu_x_OS-typical chiral signals endowed by the chiral Pen through bio-to-nano chirality transfer, confirming the successful formation of Cu_x_OS NPs. (Fig. S11). Notably, the broad absorption peak of the acceptor Cu_x_OS centered at 520 nm overlapped with the UCL of the UCNPs donor in the visible region (Fig. 2e), providing a theoretical basis for the effective quenching of the luminescence of UCNPs by Cu_x_OS.

For the construction of UCNPs/Cu_x_OS@ZIF nanoprobes, UCNP-OA should first be hydrophilic modified using polyvinylpyrrolidone (PVP) since the self-assembly of ZIF-8 was carried out in methanol. FT-IR spectroscopy showed that the -CH_2_- stretching vibration peaks representing the OA ligand at 2853 cm^−1^ and 2926 cm^−1^ disappeared after the OA ligand being removed by hydrochloric acid. Then, the characteristic signals of PVP at 2955 cm^−1^, 1656 cm^−1^, 1425 cm^−1^ and 1292 cm^−1^ appeared, indicating the successful modification of PVP on UCNPs (Fig. 2f). After the pretreatment, UCNPs and Cu_x_OS were encapsulated with ZIF-8 to form UCNPs/Cu_x_OS@ZIF chiral nanoprobes at room temperature. By adjusting the additive amount of UCNPs, we synthesized two sizes of UCNPs/Cu_x_OS@ZIF (Fig. S12). For consideration of subsequent cell and in vivo experiments, we chose the smaller size of UCNPs/Cu_x_OS@ZIF as our sensing probe. The TEM image showed that the overall size of the designed nanoprobe was 163.85 ± 11.68 nm, with uniform morphology and good dispersion (Fig. 2c). UCNPs/Cu_x_OS were effectively incorporated into the ZIF network, as evidenced by the EDS element mappings of Yb, Y, Zn, Cu, and S (Fig. 2d). The XRD patterns showed that the diffraction peaks of ZIF-8 still existed after the construction of UCNPs/Cu_x_OS@ZIF (Fig. S13). As can be seen from Fig. S14, due to the presence of Cu_x_OS, the designed nanoprobes exhibited a broad absorption trend similar to that of Cu_x_OS, resulting in a weak UCL signal. The quenching efficiency of UCL was calculated to be 94% (Fig. 2g). Besides, under 980 nm excitation, the luminescence lifetime of ^4^S_3/2_ state of Er^3+^ in UCNPs donors decreased significantly from 219 μs to 161 μs, confirming the FRET process (Fig. S15). In addition, since the chiral activity of the nanoprobe was derived from Cu_x_OS, its CD spectrum was consistent with that of Cu_x_OS, whereas UCNPs@ZIF was devoid of any chirality (Fig. 2h).

### H_2_S sensing in aqueous solution

As a vital component in the entire sensing system, in the presence of H_2_S, which was produced by hydrolysis of Na_2_S in water, the Cu^2+^ ions in Cu_x_OS will be reduced to Cu^+^, as shown in the following equation:

$${{\rm{Cu}}}_{{\rm{x}}}{\rm{OS}}({{\rm{Cu}}}^{2+})+{{\rm{H}}}_{2}{\rm{S}}\to {{\rm{Cu}}}_{{\rm{x}}}{\rm{S}}({{\rm{Cu}}}^{+})+{{\rm{H}}}_{2}{\rm{O}}$$

**Fig. 3:**
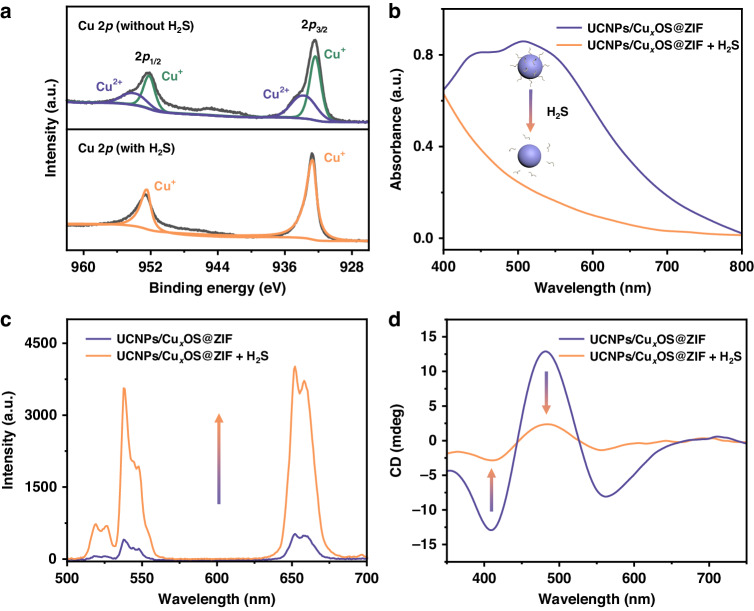
Changes of properties of composite nanoprobes before and after reaction with H_2_S. **a** XPS analysis of Cu 2p of Cu_x_OS NPs before and after reaction with H_2_S. Changes of (**b**) absorption spectra, **c** UCL spectra and (**d**) CD spectra of UCNPs/Cu_x_OS@ZIF nanoprboes before and after the reaction with H_2_S

The X-ray photoelectron spectroscopy (XPS) confirmed the change of valence state of Cu in Cu_x_OS (Fig. 3a). In XPS spectral of the synthesized Cu_x_OS, the two obvious main peaks were from Cu^+^ with binding energies (BEs) of 932.7 eV and 952.5 eV, respectively, and the other two specific peaks with BEs of 933.3 eV and 952.9 eV belong to Cu^2+^. After the reaction of Cu_x_OS with H_2_S, only two peaks with BEs of 932.7 eV and 952.5 eV remained in the spectrum, indicating that the Cu^2+^ in Cu_x_OS was reduced to Cu^+^ by H_2_S. The above reaction between Cu_x_OS and H_2_S led to a series of changes in the properties of UCNPs/Cu_x_OS@ZIF nanoprobes. Due to the reduction of Cu_x_OS, the absorption signal of the nanoprobes decreased significantly (Fig. 3b), which further led to a significant recovery of the UCL of the nanoprobes (Fig. 3c). In addition, the chirality of the nanoprobes was also significantly reduced due to the destruction of Cu_x_OS (Fig. 3d). The dual UCL/CD response of nanoprobes to H_2_S is the basis for subsequent detection.

**Fig. 4:**
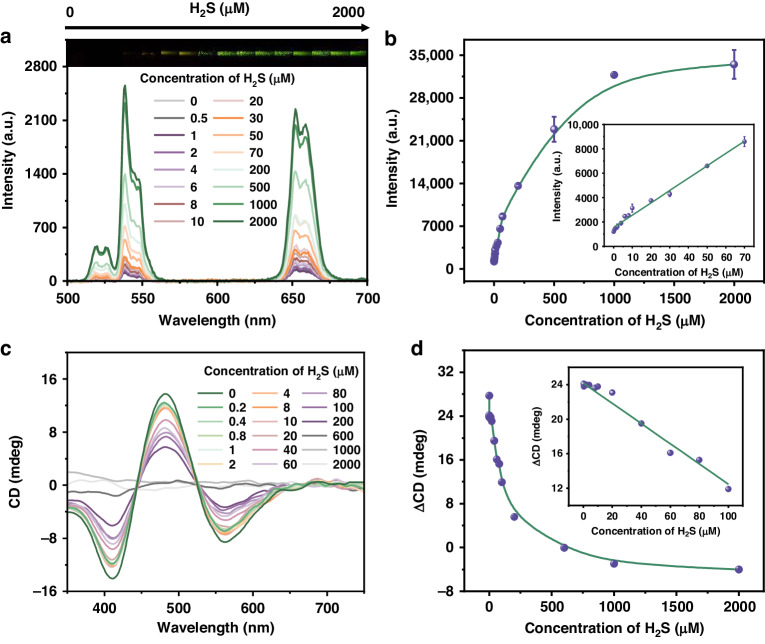
H_2_S sensing in aqueous solutions. **a** UCL spectra of UCNPs/Cu_x_OS@ZIF nanoprobes with the addition of H_2_S. Inset shows the luminescence changes of the corresponding solution under 980 nm laser irradiation. **b** Plot of the UCL intensity against the concentration of H_2_S. Inset shows the linear relationship of UCL intensity versus H_2_S concentration. **c** CD spectra of UCNPs/Cu_x_OS@ZIF nanoprobes with the addition of H_2_S. **d** Plot of the ΔCD (CD_481_-CD_411_) against the concentration of H_2_S. Inset shows the linear relationship of ΔCD versus H_2_S concentration

Figure 4 shows the UCL/CD dual response of UCNPs/Cu_x_OS@ZIF nanoprobes to H_2_S in an aqueous solution. Figure 4a illustrates the luminescence of UCNPs/Cu_x_OS@ZIF after reacting with different concentrations of H_2_S under the excitation of 980 nm. From the inset photo of Fig. 4a, it can be clearly seen that the green luminescence recovered gradually with the increase of H_2_S concentration. We recorded the UCL spectra and the corresponding intensity of nanoprobes reacting with 0-2 mM H_2_S (Fig. 4a, b). When the concentration of H_2_S increased from 0 to 70 μM, the UCL intensity had a linear relationship with the concentration of H_2_S, as shown in the inset of Fig. 4b. The limit of detection (LOD) was calculated as 160 nM (3σ/s, where σ is the standard deviation of blank signal based on 20 individual detections and s is the slope of the calibration curve). The CD signal was also monitored (Fig. 4c, d). With the increase of H_2_S concentration, the chiral Cu_x_OS was gradually reduced to the achiral Cu_x_S. The CD signal was weakened and linearly correlated with H_2_S concentration in the range of 0–100 μM, with a LOD of 66 nM. It was noteworthy that the ZIF-8 shell did not suffer significant etching after the addition of H_2_S, which was critical in the subsequent selective testing (Fig. S16).

**Fig. 5:**
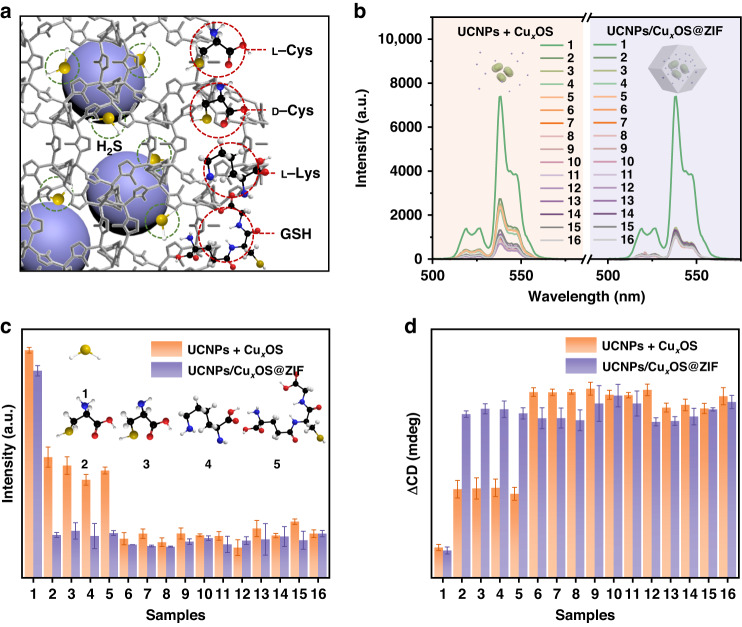
Influence of ZIF-8 shell on probe detection selectivity. **a** Schematic diagram of ZIF-8 screening for hydrogen sulfide gas molecules. **b** UCL spectra and (**c**) the corresponding UCL intensity of control and experiment group reacting with different interfering substances (1 mM): (1) H_2_S, (2) L-Cys, (3) D-Cys, (4) L-Lys, (5) GSH, (6) Glu, (7) Gly, (8) Phe, (9)Ala, (10) Glucose, (11) AA, (12) H_2_O_2_, (13) KCl, (14) NaCl, (15) Na_2_SO_4_, (16) Blank. **d** The ΔCD of control and experiment group reacting with different interfering substances (1 mM)

As a part of the nanoprobes, ZIF-8 not only stabilizes Cu_x_OS and UCNPs, but more importantly, its unique pore structure gives it the characteristic of gas molecular sieve^29^. In short, H_2_S molecules can easily enter the interior of ZIF-8, while other molecules were blocked, as shown in Fig. 5a. To verify the role of ZIF-8, 14 disruptors were selected to test the selectivity of the nanoprobes. A mixture of Cu_x_OS and UCNPs without encapsulated ZIF-8 was used as the probe in the control group, while UCNPs/Cu_x_OS@ZIF nanoprobe was used in the experimental group. After the reaction, UCL spectra and the intensity histograms of the two groups were recorded (Fig. 5b, c). It can be found that most substances do not cause UCL recovery, but L/D-Cys, L-Lys, and GSH in the control group could restore UCL to a certain extent, indicating that these substances could contact and react with Cu_x_OS, which was very unfavorable for the specificity of detection. Surprisingly, in the experimental group, no substance except H_2_S could cause the recovery of the UCL, confirming that ZIF-8 could effectively separate interfering molecules such as L/D-Cys, L-Lys and GSH. Correspondingly, the CD spectra and the intensity histogram of the two groups also showed that only H_2_S molecules could reduce the CD signals of the nanoprobe, as shown in Figs. 5d and S17. These results demonstrate that our designed UCNPs/Cu_x_OS@ZIF nanoprobes have excellent selectivity for H_2_S.

### H_2_S sensing at the living cells

**Fig. 6:**
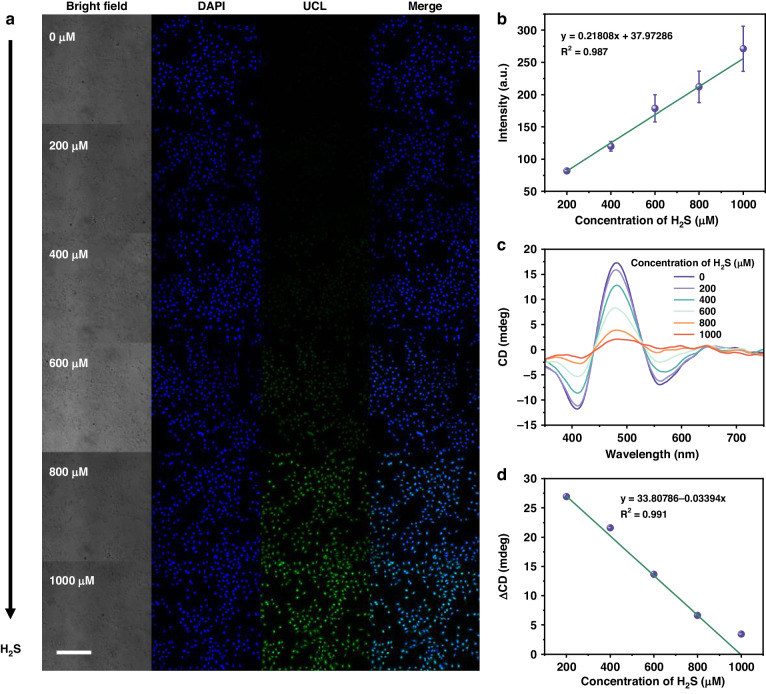
H_2_S sensing in living cells. **a** Confocal images of UCNPs/Cu_x_OS@ZIF-pretreated Hela cells reacting with various H_2_S (0–1 mM). **b** The corresponding linear relationship of UCL intensity versus H_2_S concentration. **c** The CD spectra and **d** the corresponding linear relationship of ΔCD of UCNPs/Cu_x_OS@ZIF-pretreated Hela cells reacting with various H_2_S (0–1 mM). Scale bar = 200 µm

H_2_S is a significant endogenous gas signaling molecule in biological systems and is involved in a variety of physiological processes. In recent years, increasing evidence has shown that endogenous H_2_S is closely related to a variety of cancers and can be used as a biomarker for cancer^30^. Therefore, it would be of great significance if the UCNPs/Cu_x_OS@ZIF nanoprobes could be applied to cells or even in vivo. The MTT assay was used to verify the biosafety of the nanoprobe. Hela cells, B16 cells, and C2C12 cells were selected, and the result in Fig. S18 revealed that the nanoprobe exhibited high cell survival even at the concentration of 200 μg mL^−1^. On the other hand, the biosafety of the nanoprobe to the three types of cells also reflected the universality of the material. Then, we demonstrated the ability of the nanoprobe responds to exogenous H_2_S in Hela cells. All groups of cells were first incubated with 100 μM NEM (a H_2_S scavenger) to remove endogenous H_2_S, and then the nanoprobes were added for incubation. Finally, a series of concentrations of Na_2_S solution was added to mimic the exogenous H_2_S environment in the cell. Confocal imaging (Fig. 6a) showed that the UCL gradually increased as the H_2_S concentration increased from 0 to 1000 μM (DAPI was used for cell localization), due to the reduction of Cu_x_OS by H_2_S. Figure 6b showed that the UCL intensity had a linear relationship with H_2_S concentration (the UCL intensity was obtained by integrating the fluorescence luminance of confocal images and the diagram of the calculation method is shown in Fig. S19), and the LOD was calculated to be 43 μM. Before the intracellular CD detection, cells were treated in the same way as in UCL detection, and blown off the wall with a pipette gun. The resulting cell suspension (2 mL) was used to detect CD signals. As shown in Fig. 6c, the CD signals decreased continuously with the increase of exogenous H_2_S. The linear relationship between the concentration of H_2_S and the corresponding ΔCD was shown in Fig. 6d, with a LOD of 22 μM. Although the designed UCNPs had been effective in improving the FRET efficiency between the donor and the acceptor, the sensing method based on the direct detection of analyte by CD signal change still had a higher sensitivity.

### In vivo imaging

**Fig. 7:**
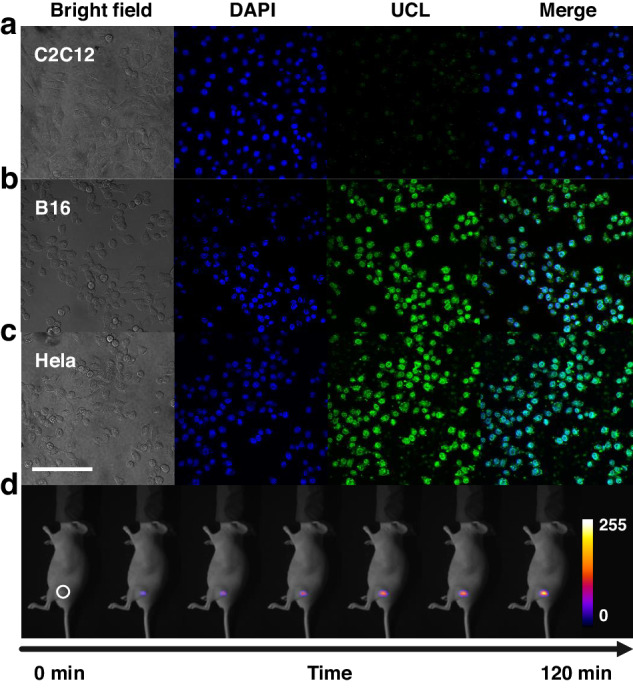
The application of UCNPs/Cu_x_OS@ZIF nanoprobes in vivo. **a**–**c** Confocal images of C2C12 cells, B16, and HeLa cells with UCNPs/Cu_x_OS@ZIF. Scale bar = 200 µm. **d** Images of tumor-bearing mouse at different times after UCNPs/Cu_x_OS@ZIF injection under 980 nm laser irradiation

Before applying UCNPs/Cu_x_OS@ZIF in vivo, the response of the designed nanoprobe to H_2_S was first tested in normal C2C12 cells as well as in two tumor cells (B16 and Hela). Figure 7a, c shows that the probe had a distinct UCL emission only in tumor cells. This was due to the overexpression of cystathionine β synthase (CBS) in tumor cells, which produced more H_2_S than in normal cells^31^. This also indicated the potential application of the nanoprobe in tumor cell identification. We then used Hela cells to establish a tumor-bearing mouse model. In Fig. 7d, 10 min after the injection of the nanoprobe, the tumor began to produce weak UCL. Within 1.5 h, the endogenous H_2_S in tumor kept destroying Cu_x_OS, and thus the luminescence intensity of the nanoprobe continued to increase. After 1.5 h, the UCL intensity remained almost unchanged due to the Cu_x_OS in the nanoprobes being completely consumed by H_2_S. In addition, the H&E-stained sections of major organs (heart, liver, spleen, lung, and kidney) of UCNPs/Cu_x_OS@ZIF-treated mice showed no abnormalities, indicating that the chiral nanoprobe was not significantly toxic to mice (Fig. S20). These results indicate that UCNPs/Cu_x_OS@ZIF can be successfully applied to in vivo H_2_S imaging.

## Discussion

In conclusion, we designed and constructed a chiral UCNPs/Cu_x_OS@ZIF dual-mode nanoprobe for highly selective and sensitive detection of H_2_S in vitro and in vivo. The excited-state energy distribution-modulated upconversion nanostructure NaYbF_4_@NaYF_4_: Yb, Er was explored as donor and UCL signal source. 100% of the sensitizer Yb^3+^ ions are integrated in the core to maximize the harvesting of NIR excitation energy, and emitter Er^3+^ ions are confined to the surface layer to effectively shorten the energy transfer distance. Chiral Cu_x_OS NPs were used as energy acceptors and CD signal providers. Subsequently, ZIF-8 was utilized to encapsulate UCNPs and Cu_x_OS as shells with enrichment and sorting advantages that improved the specificity of the detection. Based on the UCL and CD signals of the designed nanoprobe in response to H_2_S, dual-mode quantitative analysis of H_2_S in an aqueous solution could be realized with detection limits of 160 nM and 66 nM, respectively. The CD signal made the probe highly sensitive compared with fluorescence detection. Notably, this nanoprobe could also be successfully applied for quantification and imaging of H_2_S in living cells and direct tracking of H_2_S levels in tumor-bearing animals. The independent functions and mutual synergies of the components in chiral nanocomposite are highly promising for applications in biomedical sensing, diagnosis, and therapy. The development of multifunctional chiral nanocomposite structures is still an emerging, challenging, and fascinating field that deserves more in-depth research.

## Materials and methods

### Materials

Erbium (III) chloride hexahydrate (ErCl_3_·6H_2_O, 99.9%), yttrium chloride hexahydrate (YCl_3_·6H_2_O, 99.9%) and ytterbium(III) chloride hexahydrate (YbCl_3_·6H_2_O, 99.9%) were purchased from Sigma-Aldrich. 1-octadecene (1-ODE, 90%), oleic acid (OA, 90%), hydroxylamine hydrochloride (HONH_3_Cl, 98%), sodium sulfide (Na_2_S, 90%) and 2-methylimidazole were purchased from Energy Chemical. Ammonium fluoride (NH_4_F, 97%), D-penicillamine (D-Pen, 98%), cupric chloride (CuCl_2_, 98%), polyvinylpyrrolidone (PVP, Mw=40 000), sodium hydroxide (NaOH, 98%), and zinc nitrate hexahydrate (Zn(NO_3_)_2_·6H_2_O, 99%) were purchased from Aladdin.

### Instruments

The crystal structures of all the synthesized nanomaterials were measured by Rigaku wide-angle X-ray diffraction (XRD) in the angular range of 2-88°. The detailed structure and morphology of the synthesized nanomaterials were obtained by JEM-2100 transmission electron microscope (TEM). The UC emission spectra were measured by QuantMaster 8000 Fluorescence Spectrometer (HORIBA Scientific). The UV-vis spectrum was recorded with a spectrophotometer (UV-2550, Shimadzu, Japan). The CD spectra was measured by MOS-450 circular dichroism spectrometer of BioLogic company of France. The Fourier Transform Infrared spectroscopy (FT-IR) was measured with the Nicolet iS10 FT-IR spectrometer (Thermo Fisher Scientific, the United States). The excitation source is 980 nm semiconductor lasers (All purchased from Changchun New Industries Co., Ltd., China). X-ray photoelectron spectroscopy (XPS) was collected by ESCALAB 250 XI electron spectrometer (Thermo). The cell Confocal images were obtained using an two-photon Nikon A1RMP microscope.

### Synthesis of NaYbF_4_ core nanoparticles

OA (6 mL), 1-ODE (15 mL), and YbCl_3_·6H_2_O (1 mmol) were mixed into a three-necked flask and heated to 160 °C. When the solid material in the solution was completely dissolved, let the solution naturally cool to 50 °C and add 5 mL methanol containing NaOH (2.5 mmol) and 5 mL methanol containing NH_4_F (4.0 mmol) in turn, heated to 70 °C to remove methanol. Afterwards, the mixture was heated to 315 °C and kept stirring for 60 min. After the mixture was cooled to room temperature, the NaYbF_4_ nanoparticles were precipitated by acetone, collected by centrifugation (6 000 rpm for 6 min) and redisperse in cyclohexane. Note that Argon gas was kept flowing during the whole experiment process.

### Synthesis of NaYbF_4_@NaYF_4_:Yb,Er core-shell nanoparticles (UCNP-OA)

OA (3 mL), 1-ODE (7 mL), YbCl_3_·6H_2_O (0.1 mmol), ErCl_3_·6H_2_O (0.01 mmol), and YCl_3_·6H_2_O (0.39 mmol) were added into a three-necked flask, heated to 160 °C and stirred until the solid material in the solution was completely dissolved. As the solution was cooled to 50 °C, 3 mL methanol containing NaOH (1.25 mmol) and 3 mL methanol containing NH_4_F (2 mmol) was added in turn and then heated to 70 °C to remove methanol. When the solution was cooled to 50 °C, 0.5 mmol of previously prepared core nanoparticles was added and heated to 85 °C to remove the cyclohexane. The mixture was then heated up to 315 °C and reacted for 60 min. After the reaction, the NaYbF_4_@NaYF_4_:Yb,Er nanoparticles were precipitated by acetone, collected by centrifugation (6 000 rpm for 6 min) and redisperse in cyclohexane. Note that Argon gas was kept flowing during the whole experiment process.

### Synthesis of PVP-stabilized NaYbF_4_@NaYF_4_:Yb,Er core-shell nanoparticles (UCNP-PVP)

2 mL cyclohexane dispersed with 0.25 mmol UCNP-OA was added into 2 mL (0.1 M) of dilute HCl solution and stirred overnight to remove the oleic acid ligands. The ligand-free nanoparticles were precipitated by adding acetone, collected by centrifugation, and dispersed in 2 mL ethanol. Then 1 mL ligand-free nanoparticles solution and 5 mL of ethanol containing PVP (0.3 g, Mw=40 000) were mixed and kept stirring for 24 h. After that, the UCNP-PVP were precipitated with moderate hexane, collected by centrifugation (10 000 rpm for 8 min), washed with ethanol and then dispersed in methanol.

### Synthesis of Cu_x_OS NPs

100 µL CuCl_2_ (0.2 mol L^−1^), 100 µL NaOH (0.4 mol L^−1^), 150 µL D-pen (0.4 mol L^−1^) and 300 µL HONH_3_Cl (1 mmol) were added into 3 mL of deionized water and stirred for 5 min to form Cu_x_OS NPs. After the reaction, the Cu_x_OS NPs were precipitated with ethanol and collected by centrifugation (10 000 rpm for 8 min). Then, the prepared Cu_x_OS nanoparticles were dispersed in water.

### Synthesis of UCNPs/Cu_x_OS@ZIF nanoprboes

4 mL methanol containing Zn(NO_3_)_2_·6H_2_O (15.191 mg) and 4 mL methanol containing 2-methylimidazole (7.125 mg) were first mixed and stirred for 1 min. Then 50 µL UCNP-PVP (30 mM) was injected. After the solution turns slightly white, 200 µL of previously prepared Cu_x_OS nanoparticles were added and keep for 30 min without disturbing. After the reaction, the UCNPs/Cu_x_OS@ZIF nanoprobes were formed and precipitated with methanol (6 000 rpm for 8 min).

### UCL/CD sensing of H_2_S

The preparation and quantification of standard H_2_S solution followed the previous reports^32^. To simulate the existence of H_2_S in physiological conditions, H_2_S standard solution was prepared by adjusting the pH of freshly formulated Na_2_S solution to neutral (pH = 7.0). All H_2_S standard solutions were hermetically stored at 4 °C and used within 60 min after formulation. To set up the standard curve for H_2_S detection, the standard H_2_S solution with various final concentrations were mixed with the same amount of UCNPs/Cu_x_OS@ZIF solution to produce 2 mL of the mixture to be tested. The mixture was incubated at room temperature under gently shaking for 10 min after that the UCL and CD signals were measured.

### Cytotoxicity evaluation

Hela, B16 and C2C12 cells were seeded in a 96-well plate and incubated for 24 h. Then different concentrations of UCNPs/Cu_x_OS@ZIF nanoprobe (25, 50, 100, 150, 200 μg mL^−1^) were added to the medium and incubated at 37 °C for 12 h. Using a standard MTT test, the cell survival rate was de-termined as a percentage of viable cells after the treatment with the nanoprboes compared to untreated cells.

### Imaging and detection in cells

Before cell imaging, Hela cells were incubated in 35-mm glass-bottom Petri dishes for 24 h. Then, the culture medium containing certain concentrations of UCNPs/Cu_x_OS@ZIF nanoprboes (200 μg mL^−1^) were added and further incubated for 3 h. The cells were then washed with HBSS (Hank’s Balanced Salt Solution) before adding different concentrations of Na_2_S solution incubating for another 1 h. Finally, the cells were collected for the determination of confocal imaging.

### Imaging in mice

For imaging in mice, 1 × 10^6^ HeLa cells suspended in 100 µL of PBS and subcutaneously injected at an indicated location into female nude mice. Three weeks after implantation, UCNPs/Cu_x_OS@ZIF NPs (in PBS) were injected subcutaneously into the tumor. Images of the anesthetized mice exposed to a 980 nm laser were recorded at various times after probe injection using an in vivo imaging system.

### Supplementary information

Supplementary information
