# Supplementary material for: Upconversion-based chiral nanoprobe for highly selective dual-mode sensing and bioimaging of hydrogen sulfide in vitro and in vivo
Source: Light Sci Appl. 2024 Aug 1;13:180. doi: 10.1038/s41377-024-01539-6 (PMC11294450; doi:10.1038/s41377-024-01539-6)
Supplement: Supplementary file 1 — Supplementary information [file 41377_2024_1539_MOESM1_ESM.docx]

**Supplementary Information for**

**Upconversion-based chiral nanoprobe for highly selective dual-mode sensing and bioimaging of hydrogen sulfide in vitro and in vivo**

Yang Lu ^1^, Xu Zhao ^1^, Dongmei Yan ^2^, Yingqian Mi ^2^, Peng Sun ^1^, Xu Yan ^1,*^,

Xiaomin Liu ^1,*^ and Geyu Lu ^1,*^

^1^ State Key Laboratory of Integrated Optoelectronics, College of Electronic Science and Engineering, Jilin University, Changchun 130012, China

^2^ Department of Immunology, College of Basic Medical Sciences, Jilin University, Changchun 130021, China

**^*^Corresponding author:**

Xu Yan (Email: yanx@jlu.edu.cn)

Xiaomin Liu (Email: [xiaominliu@jlu.edu.cn](mailto:xiaominliu@jlu.edu.cn))

Geyu Lu (Email: lugy@jlu.edu.cn)

**
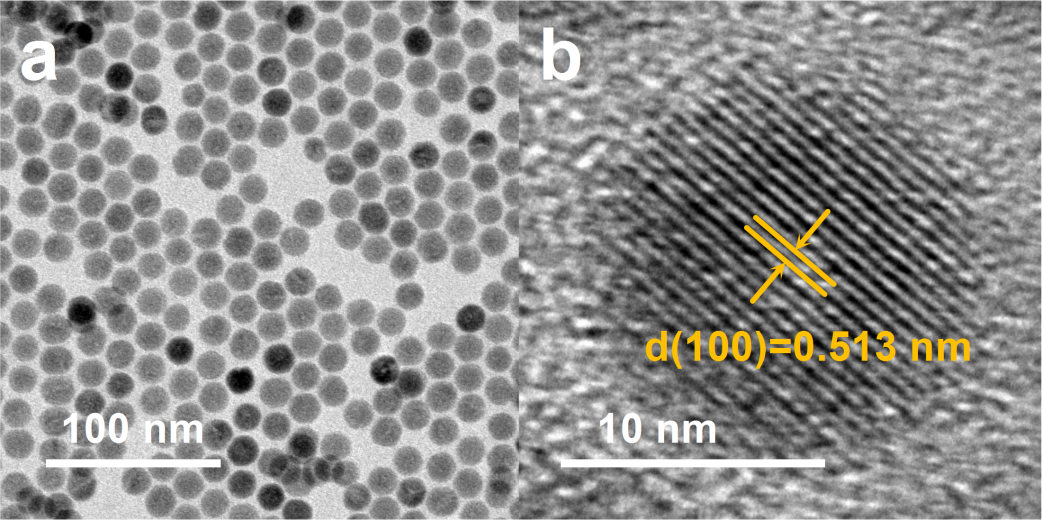
**

**Fig. S1** **a** Transmission electron microscopy (TEM) images of NaYbF_4_ core nanoparticles. **b** The high-resolution TEM image of the core nanoparticles.

**
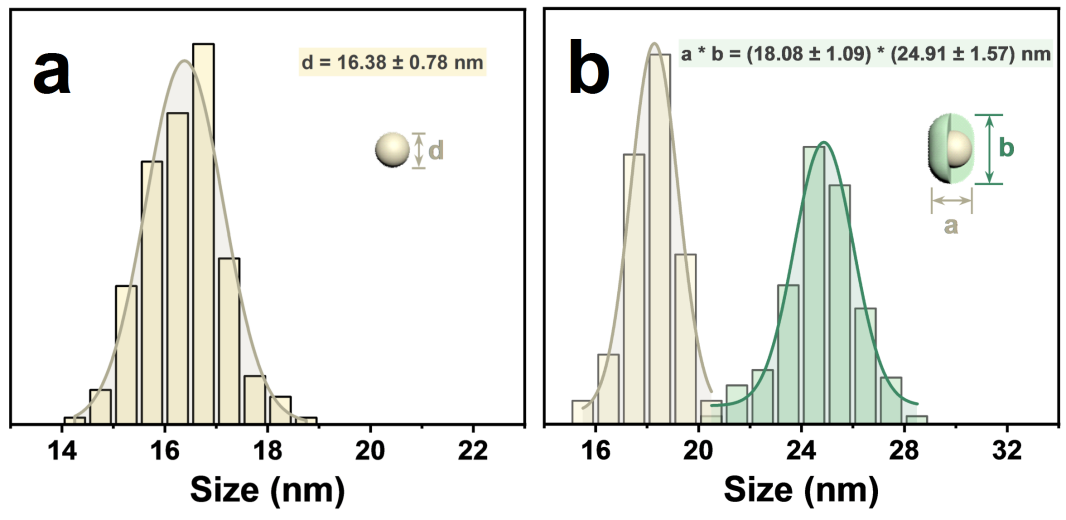
**

**Fig. S2** The size histograms of **a** NaYbF_4_ core nanoparticles and **b** NaYbF_4_@NaYF_4_:Yb,Er core-shell nanoparticles.


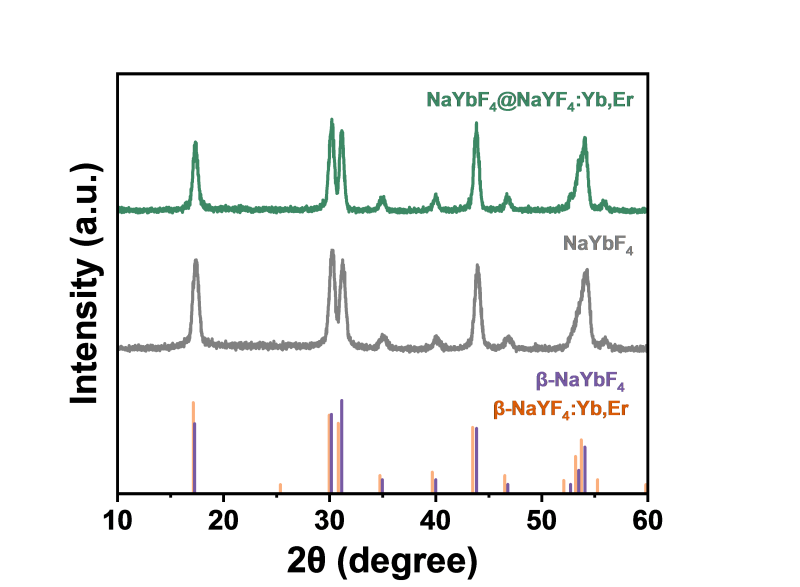


**Fig. S3** XRD patterns of NaYbF_4_ core nanoparticles, and NaYbF_4_@NaYF_4_:Yb,Er core-shell nanoparticles. (β-NaYbF_4_ JCPDS 27-1427; β-NaYF_4_:Yb,Er JCPDS 28-1192).


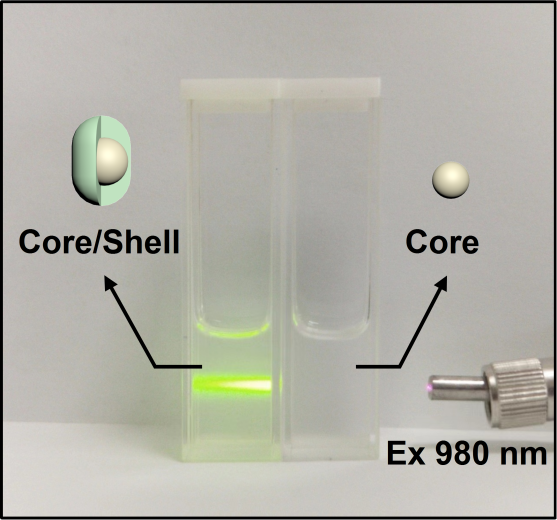


**Fig. S4** NaYbF_4_ core (right) and NaYbF_4_@NaYF_4_:Yb,Er core-shell (left) nanoparticles dispersed in cyclohexane, illuminated with 980 nm laser

**
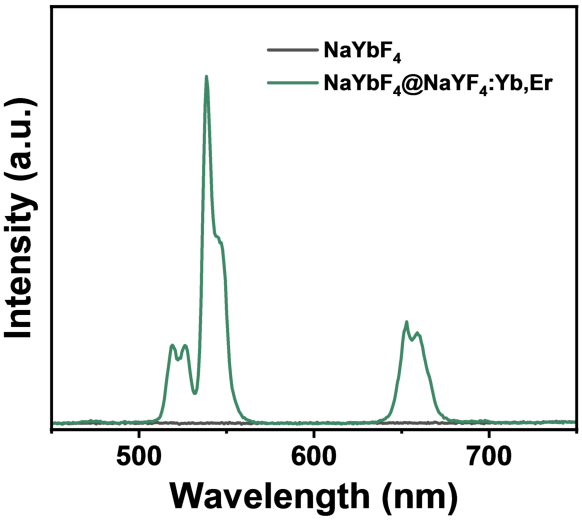
**

**Fig. S5** The UCL spectra of NaYbF_4_ core nanoparticles and NaYbF_4_@NaYF_4_:Yb,Er core-shell nanoparticles dispersed in cyclohexane, illuminated with 980 nm laser.


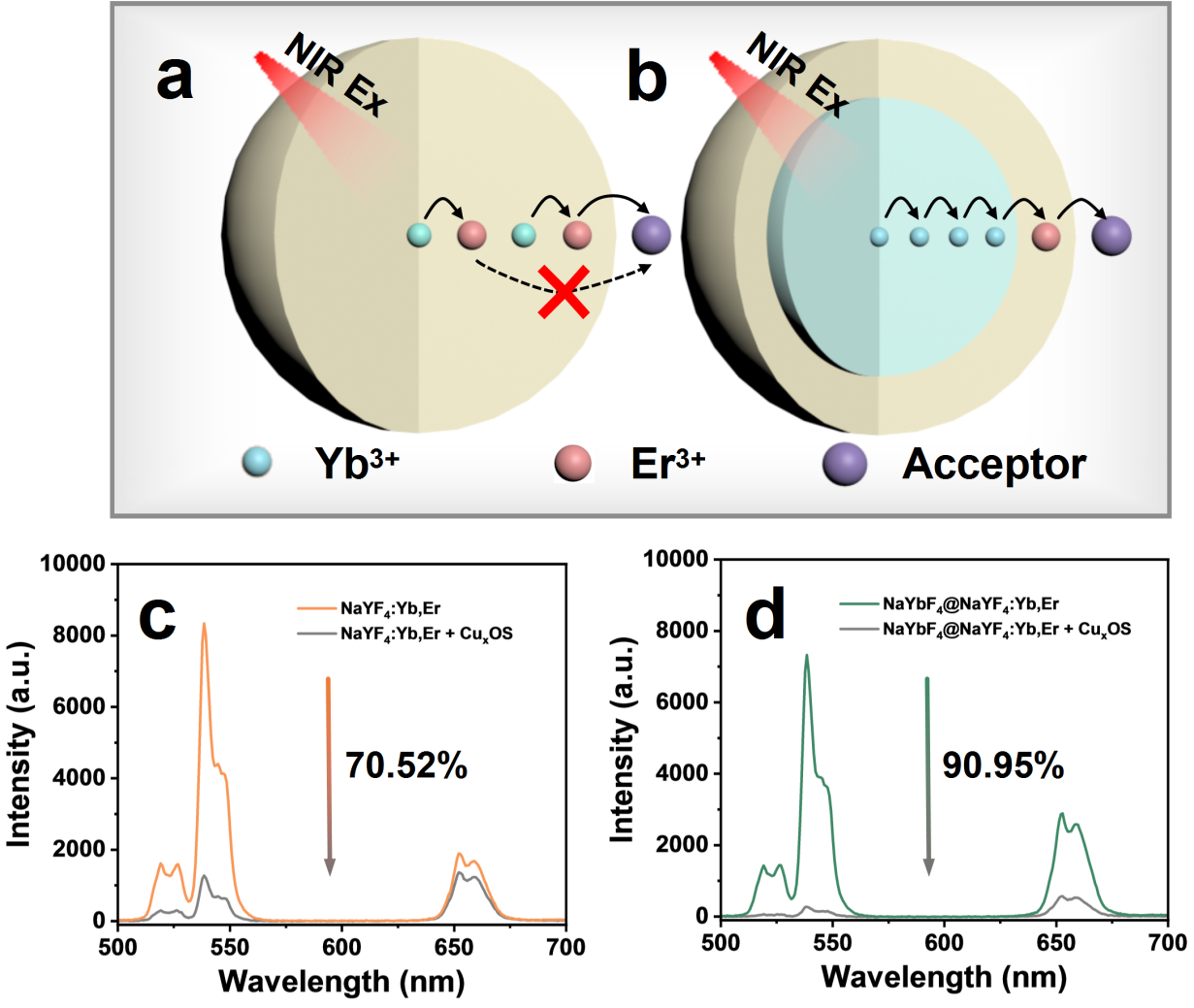


**Fig. S6** Energy transfer diagram of **a** NaYF_4_:Yb,Er and **b** NaYbF_4_@NaYF_4_:Yb,Er. The UCL spectra of **c** NaYF_4_:Yb Er nanoparticles and **d** NaYbF_4_@NaYF_4_:Yb, Er nanoparticles before and after the addition of Cu_x_OS nanoparticles.

**
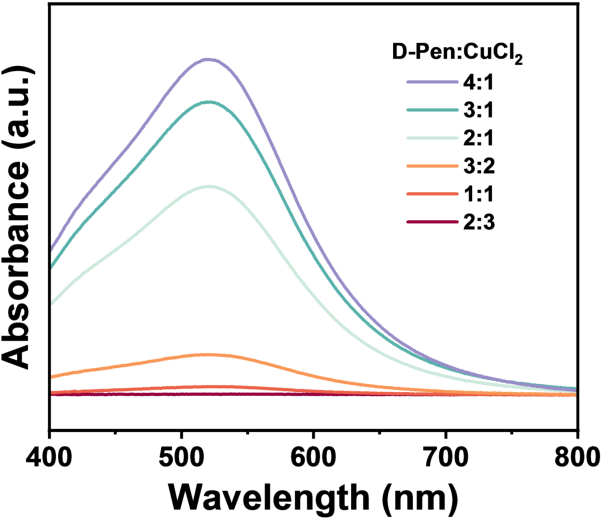
**

**Fig. S7** The absorption spectra of Cu_x_OS synthesized with different D-Pen:CuCl_2_.

**
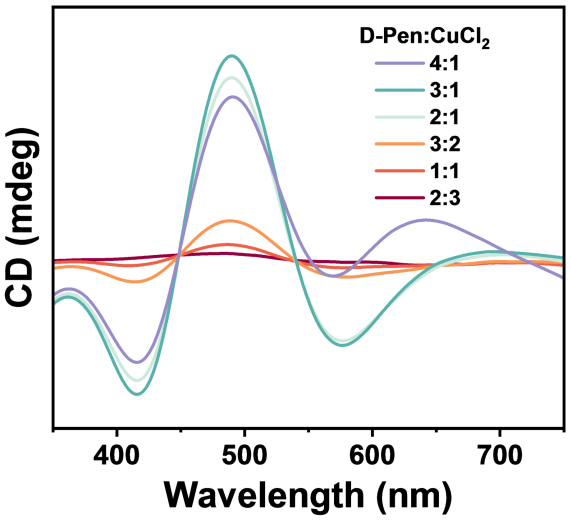
**

**Fig. S8** The CD spectra of Cu_x_OS synthesized with different D-Pen:CuCl_2_.

**
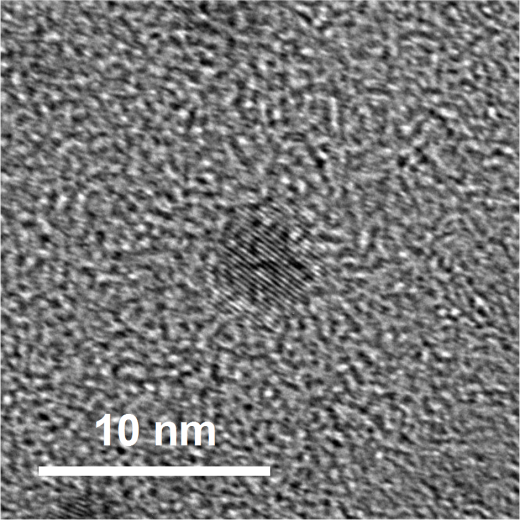
**

**Fig. S9** The high-resolution TEM image of the Cu_x_OS nanoparticles.

**
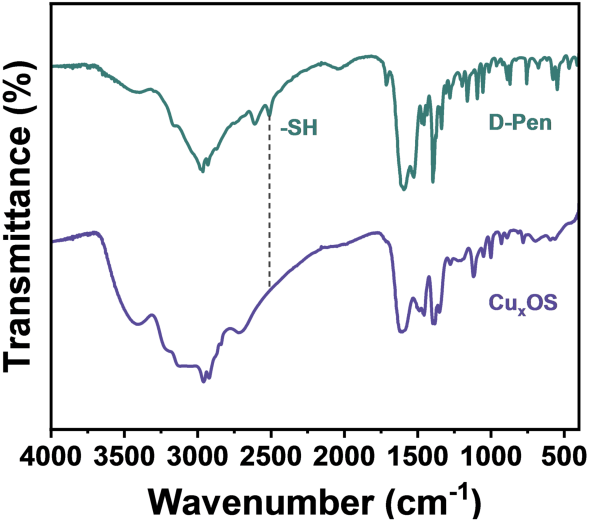
**

**Fig. S10** Fourier transform infrared spectroscopy (FT-IR) of D-Pen and Cu_x_OS.

**
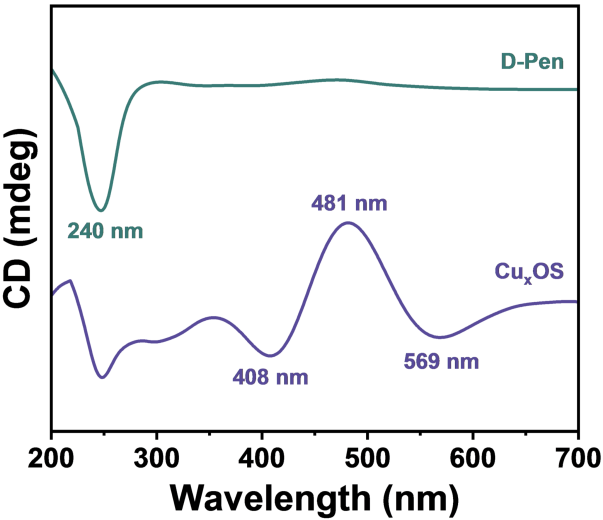
**

**Fig. S11** The CD spectra of D-Pen and Cu_x_OS.


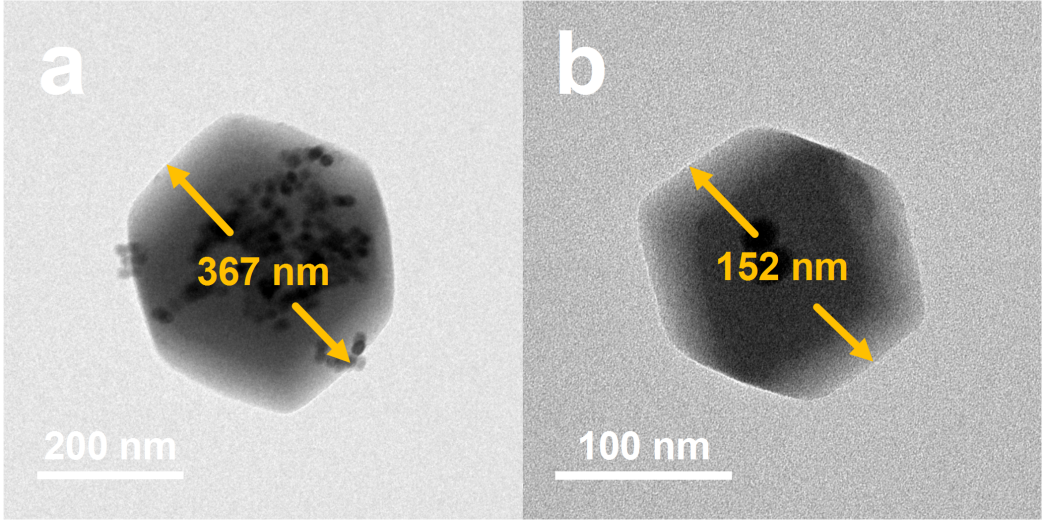


**Fig.** **S12** The TEM images of the UCNPs/Cu_x_OS@ZIF nanoprobe synthesized by adding **a** 500 μL UCNPs (30 mM) and **b** 50 μL UCNPs (30 mM).

**
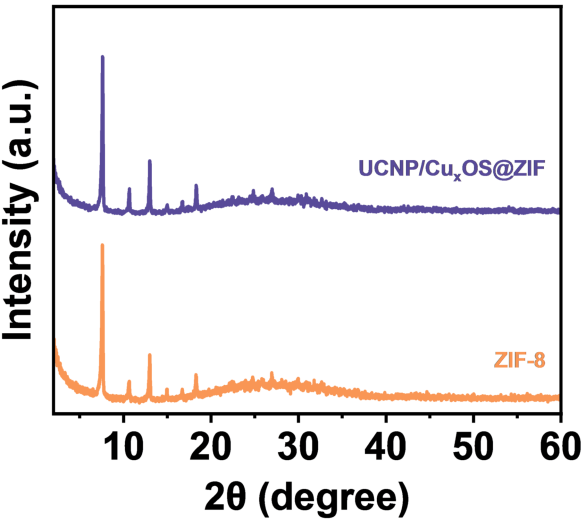
**

**Fig. S13** XRD patterns of ZIF-8 and UCNPs/Cu_x_OS@ZIF.

**
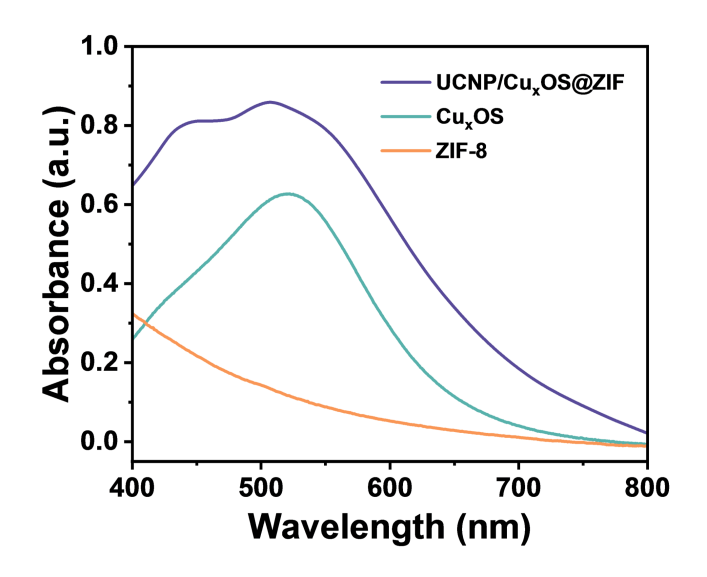
**

**Fig. S14** The absorption spectra of ZIF-8, Cu_x_OS and UCNPs/Cu_x_OS@ZIF.

**
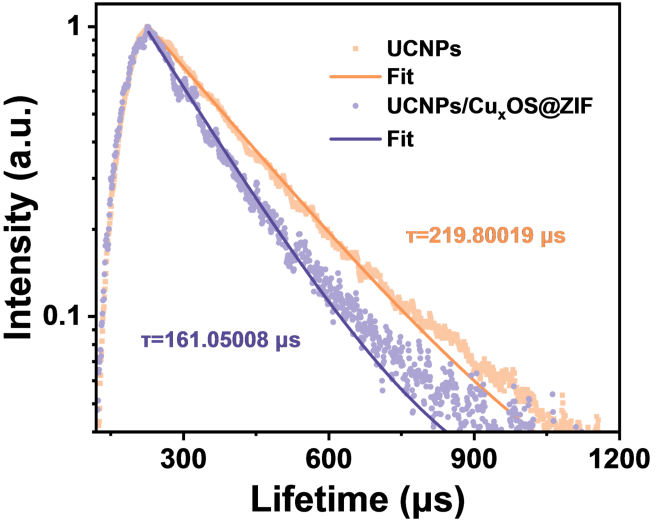
**

**Fig. S15** The luminescence decay curves and the exponential fitting of the lifetimes of Er^3+^ emissions at 545 nm before and after the formation of UCNPs/Cu_x_OS@ZIF under 980 nm excitation.

**
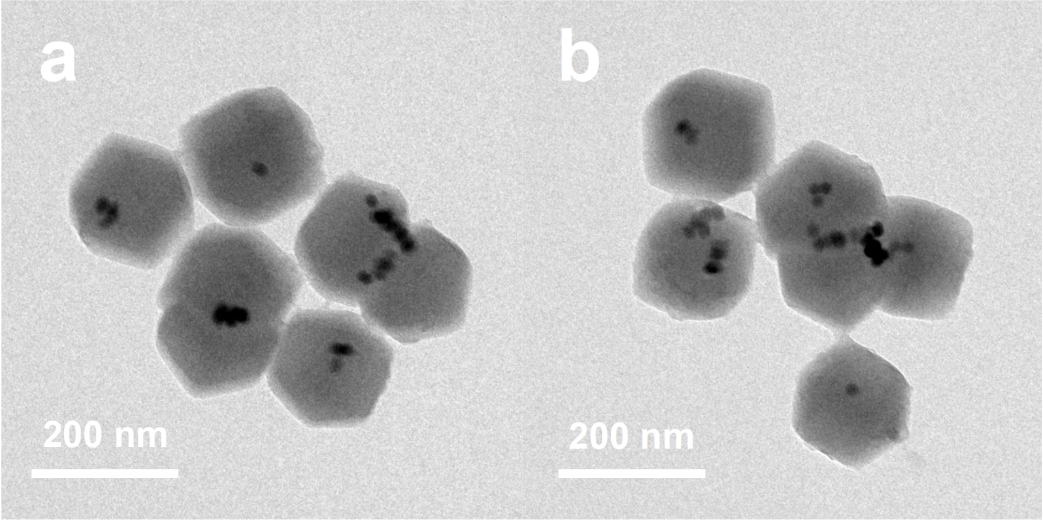
**

**Fig. S16** The TEM images of UCNPs/Cu_x_OS@ZIF before and after the reaction with H_2_S.

**
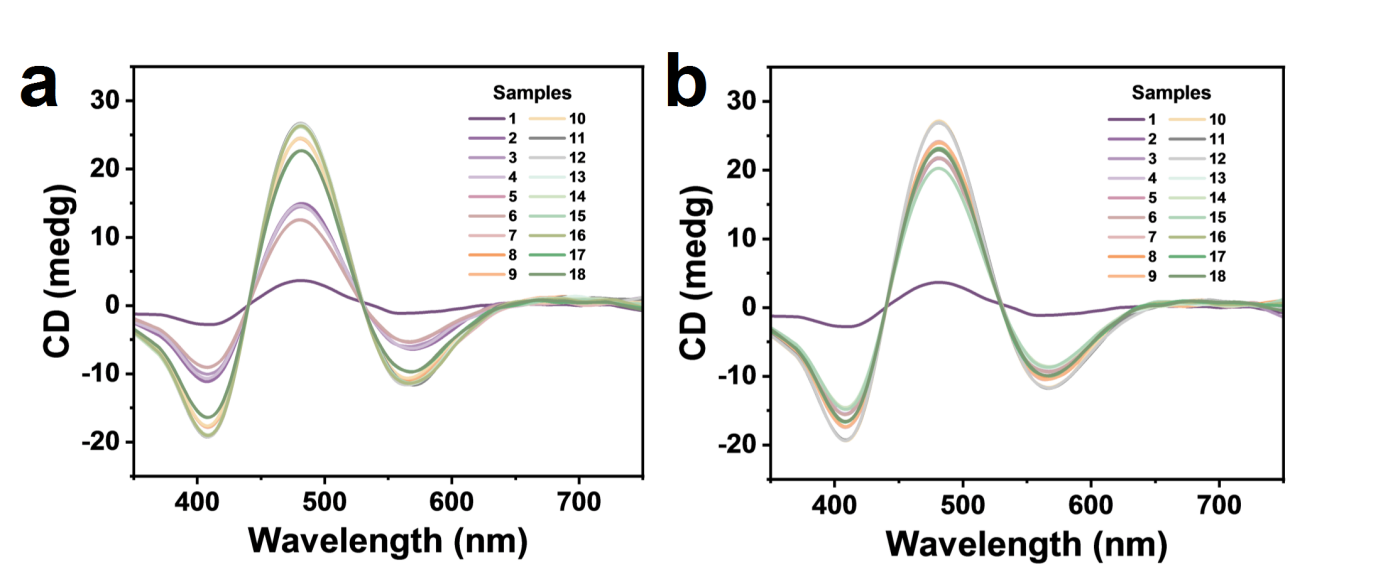
**

**Fig. S17** The CD spectra of **a** control group and **b** experimental group reacting with different interfering substances (1 mM): (1) H_2_S, (2) L-Cys, (3) D-Cys, (4) L-Lys, (5) GSH, (6) Glu, (7) Gly, (8) Phe, (9)Ala, (10) Glucose, (11) AA, (12) H_2_O_2_, (13) KCl, (14) NaCl, (15) Na_2_SO_4_, (16) Blank.

**
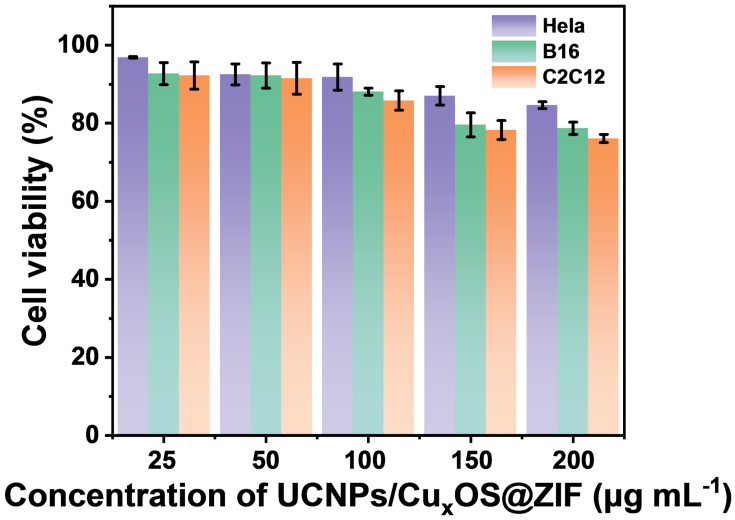
**

**Fig. S18** Survival rate of Hela cells, B16 cells and C2C12 cells incubated with different concentrations of UCNPs/Cu_x_OS@ZIF (25, 50, 100, 150 and 200 μg mL^-1^) for 24 h.


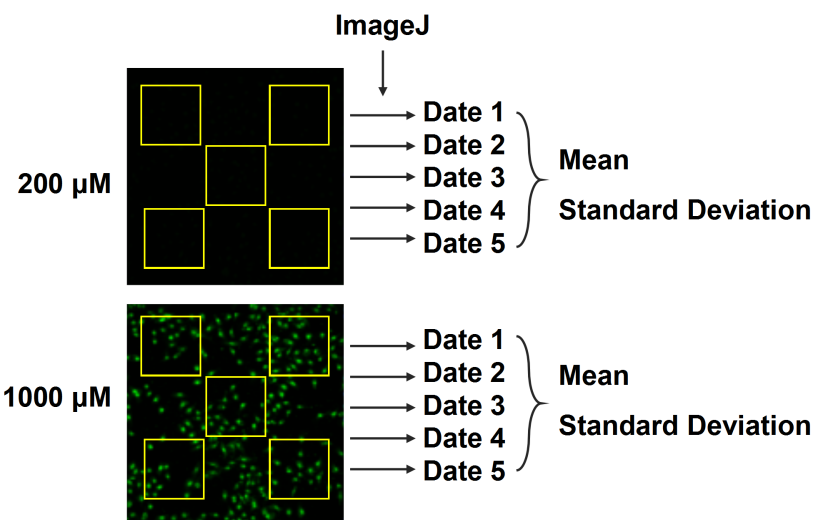


**Fig. S19** The schematic diagram of UCL intensity calculation method for the cell confocal images (the images of 200 μM and 1000 μM are used as examples).


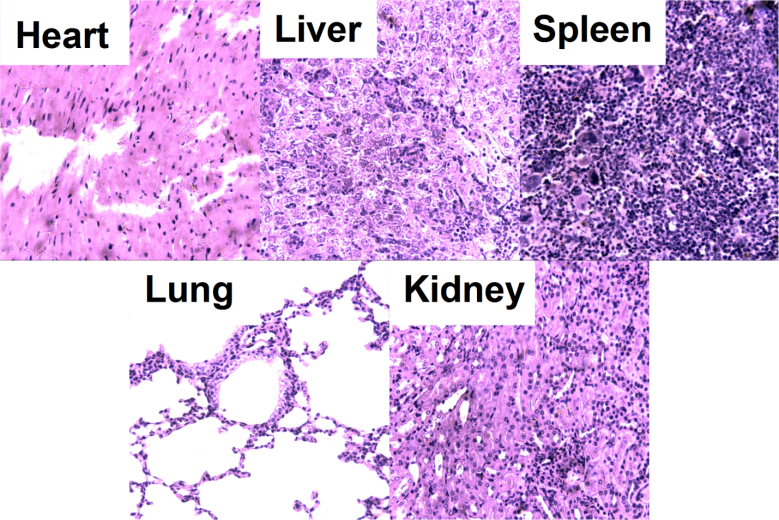


**Fig. S20** The H&E-stained sections of the major organs (heart, liver, spleen, lung, and kidney) of the UCNPs/Cu_x_OS@ZIF-treated mouse.
